# Genetic Pleiotropy and Causal Pathways Linking Glycemic Traits to Asthma: An Integrated Proteogenomic Investigation

**DOI:** 10.3390/children12111443

**Published:** 2025-10-24

**Authors:** Lin Chen, Juntao Lin, Yan Zhao, Guangli Zhang, Zhenxuan Kong, Chunlan Qiu, Kaicheng Peng, Hui Liu, Zhengxiu Luo

**Affiliations:** 1 National Clinical Research Center for Child Health and Disorders, Ministry of Education Key Laboratory of Child Development and Disorders, Chongqing Key Laboratory of Child Rare Diseases in Infection and Immunity, Children’s Hospital of Chongqing Medical University, Chongqing 400014, China; 2 Department of Respiratory Medicine, National Clinical Research Center for Child Health and Disorders, Ministry of Education Key Laboratory of Child Development and Disorders, Chongqing Key Laboratory of Child Rare Diseases in Infection and Immunity, Children’s Hospital of Chongqing Medical University, Chongqing 400014, China

**Keywords:** asthma, genetic architecture, diabetes mellitus, glycemic traits, colocalization

## Abstract

**Highlights:**

**What are the main findings?**

- Obesity (BMI) and type 2 diabetes mellitus (T2DM) show significant genetic correlations and causal effects on asthma risk.
- Shared pleiotropic loci and key proteins (e.g., IL6R, MAPK3, CSF2) link diabetes/glycemic traits with asthma through inflammatory pathways.

**What is the implication of the main finding?**

- These findings suggest that metabolic dysfunction contributes to asthma pathogenesis via shared genetic and immunological mechanisms.
- Targeting colocalized proteins and pathways such as JAK-STAT signaling may provide novel therapeutic strategies for comorbid diabetes and asthma.

**Abstract:**

**Background:** While diabetes is a recognized risk factor for asthma, the shared genetic components between diabetes/glycemic traits and asthma remain unclear. This study investigates the genetic associations, causal relationships, and underlying mechanisms linking these conditions. **Methods:** We assessed global genetic correlations using linkage disequilibrium score regression (LDSC), high-definition likelihood analysis (HDL), and genetic covariance analysis (GNOVA). Trait pairs with significant correlations subsequently underwent genetic overlap analysis (Genetic analysis integrating Pleiotropy and functional Annotation, GPA) and local genetic correlation analysis (Local Genetic Variant Association Analysis, LAVA). Cross-phenotype association (CPASSOC) and multitrait analysis of GWAS (MTAG) identified potential pleiotropic loci, followed by colocalization and functional annotation. Proteome-wide association study (PWAS) revealed proteins and pathways shared between diabetes/glycemic traits and asthma. Generalized summary-data-based Mendelian randomization (GSMR) was used to evaluate causal effects between diabetes/glycemic traits and asthma. **Results:** Significant genetic correlations were observed between body mass index (BMI) and asthma (rg = 0.280–0.397; FDR < 0.05), type 2 diabetes mellitus (T2DM) and asthma (rg = 0.240–0.289; FDR < 0.05) across all three methods. GPA revealed significant genome-wide genetic overlap, highest for BMI and asthma (pleiotropy association ratio [PAR] = 0.377) and T2DM-asthma (PAR = 0.353). LAVA identified 111 significant local correlation regions, predominantly between T2DM and asthma (70 regions). Colocalization analysis identified 24 shared pleiotropic loci, predominantly on chromosome 8. Local genetic correlation analysis revealed extensive correlations between T2DM and asthma. PWAS identified 46 shared proteins, with IL6R, MAPK3, and CSF2 being key hubs. Protein–protein interaction analysis highlighted enrichment in JAK-STAT signaling, Th1/Th2 differentiation, and IL-17 pathways. GSMR demonstrated causal effects of BMI (OR = 1.47, 95% CI: 1.42–1.53, FDR < 0.05) and T2DM (OR = 1.06, 95% CI: 1.04–1.08, FDR < 0.05) on increased asthma risk, with no evidence of reverse causality. **Conclusions:** Obesity (BMI) and T2DM exert causal effects on asthma risk via shared genetic loci and inflammatory pathways, particularly involving IL6R, MAPK3, CSF2, and JAK-STAT signaling. Targeting these colocalized proteins may offer potential therapeutic strategies.

## 1. Introduction

Asthma and diabetes mellitus (DM) are prevalent chronic diseases posing major public health burdens. Asthma is a complex heterogeneous disease caused by the combined environmental and genetic factors, characterized by airway inflammation and airway hyperresponsiveness (AHR). Despite inhaled corticosteroids (ICS) and long-acting beta-adrenoceptor agonists (LABA) providing effective control for asthma, some patients remain poorly managed [1]. DM includes type 1 diabetes mellitus (T1DM) and type 2 diabetes mellitus (T2DM). T1DM is a chronic autoimmune disease driven by genetic susceptibility, immune dysregulation, and environmental factors, leading to pancreatic β-cell destruction [2]. T2DM is a metabolic disorder characterized by insulin resistance and defective insulin secretion [3], with rising global incidence [4] influenced by genetic background and aging [5]. T1DM involves Th1-type autoimmune responses, while asthma is dominated by Th2-type allergic responses, with mutual inhibition between these pathways [6]. However, one recent meta-analysis revealed that childhood asthma is associated with increased T1DM risk (HR = 1.30, 95% CI 1.05–1.61, *p* = 0.014), whereas T1DM does not elevate asthma risk [7]. Conversely, T2DM significantly increases asthma risk [8]. Asthma subtypes diverge by onset age and atopy status [9]. On the basis of differences in immune responses and airway inflammation patterns, asthma can be classified into type 2 (T2) high or T2-low asthma [7]. Th2 asthma is primarily manifested by upregulation of the Th2 immune pathway, including elevated cytokines such as IL-4, IL-5, and IL-13, as well as eosinophilic airway inflammation [10]. This subtype is typically responsive to corticosteroid therapy, often accompanied by atopic predisposition, and represents the primary target population for current biologic therapies [11]. In contrast, non-Th2 asthma accounts for 30–50% of severe asthma cases and is characterized by neutrophilic sputum infiltration and corticosteroid resistance [12]. Growing evidence indicates that DM and asthma frequently coexist and may share certain genetic architecture and pathological mechanisms (e.g., inflammation, oxidative stress). However, research on their shared genetics remains limited, and consistency across DM types and asthma subtypes is unclear.

Integrating Genome-wide association study (GWAS) data with genetic correlation analysis, pleiotropic variant identification, and cross-trait analysis enables detailed exploration of common genetic foundations across diseases, revealing shared pathogenic genes/pathways and novel insights into complex disease mechanisms.

GWASs minimize reverse causation, lifestyle factors, or environmental confounders. Investigating shared genetic mechanisms across multiple diseases through GWAS summary data is a key genetic research method [13,14,15]. Integrating GWAS data with genetic correlation analysis, pleiotropic variant identification, and cross-trait analysis enables detailed exploration of common genetic foundations across diseases, revealing shared pathogenic genes/pathways and novel insights into complex disease mechanisms, Therefore, this study leverages GWAS data to investigate the shared genetic architecture, causal relationships, and potential functional pathways among DM, glycemic traits and asthma subtypes through genome-wide pleiotropic association analyses.

## 2. Method

### 2.1. Study Population and Design

**Figure 1:**
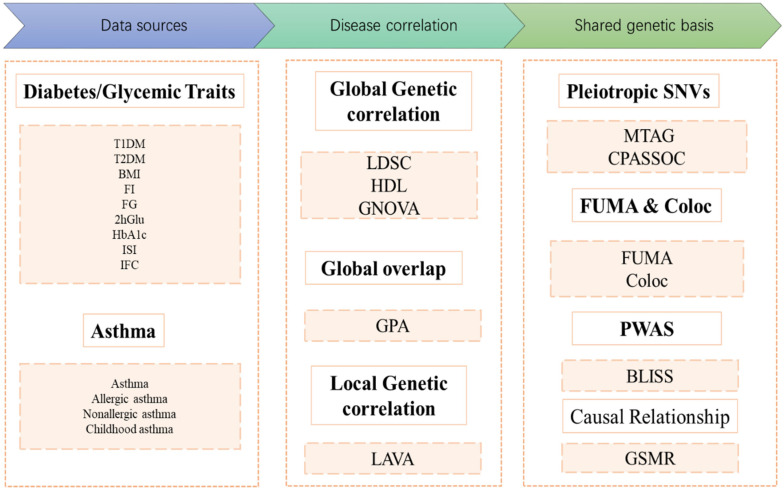
Flow diagram of the genetic analysis. COLOC: Colocalization; T1DM: Type 1 Diabetes Mellitus; T2DM: Type 2 Diabetes Mellitus; BMI: Body Mass Index; FI: Fasting Insulin; FG: Fasting Glucose; 2hGlu: 2 Hour Postprandial Glucose; HbA1c: Hemoglobin A1c; ISI: Insulin Sensitivity Index; IFC: Insulin Fold Change after Oral Glucose Tolerance Test; LDSC: Linkage Disequilibrium Score Regression; HDL: High-Definition Likelihood Analysis; GNOVA: Genetic Covariance Analyzer; GPA: Genetic analysis integrating Pleiotropy and functional Annotation; LAVA: Local Genetic Variant Association Analysis; MTAG: Multitrait Analysis of Genome-wide Association Studies; CPASSOC: Cross-Phenotype Association Analysis; PWAS: Proteome-Wide Association Study; BLISS: Biomarker Expression Level Imputation using Summary Statistics; GSMR: Generalized Summary-data-based Mendelian Randomization. FUMA: Functional Mapping and Annotation of Genetic Associations.

The study design is summarized in Figure 1. We utilized publicly available GWAS summary statistics from European-ancestry cohorts. First, global genetic correlation analyses were conducted by three distinct methods, namely, linkage disequilibrium score regression (LDSC), high-definition likelihood analysis (HDL), and genetic covariance analysis (GNOVA), to explore global genetic correlations between DM/glycemic traits and asthma. Genetic overlap analyses and local genetic correlation analyses were subsequently performed. Multitrait analysis of GWAS (MTAG), cross-phenotype association (CPASSOC), functional mapping and annotation of genetic associations (FUMA), and colocalization were employed to identify pleiotropic loci. Proteome-wide association studies (PWAS) were conducted to identify proteins significantly associated with diabetes or glycemic traits and asthma risk. Finally, the potential causal relationships between diabetes or glycemic traits and asthma were investigated with generalized summary-data-based Mendelian randomization (GSMR).

### 2.2. Data Sources

We utilized GWAS summary statistics obtained from large publicly available databases. Asthma data for different phenotypes were derived primarily from the FinnGen database. To avoid sample overlap, GWAS summary statistics for diabetes and related metabolic traits were selected from other large consortia, excluding Finnish populations, including DIAMANTE, UK Biobank, GIANT, and MAGIC. Detailed information on these GWAS summary statistics were provided in Supplementary Table S1.

### 2.3. Genetic Correlation Analysis

To evaluate the genetic architecture shared between DM/glycemic traits and asthma, we employed three analytical methods: LDSC [16], HDL [17], and GNOVA [18]. HDL and GNOVA, as complementary methods of LDSC, enable a more comprehensive assessment of genome-wide linkage disequilibrium (LD) information and improve the precision of genetic correlation estimates. For LDSC and GNOVA, the European ancestry genomic data from the 1000 Genomes Project were utilized as reference panels, whereas the UK Biobank data served as the reference panel for HDL. To ensure analytical accuracy, we filtered linkage disequilibrium scores by retaining only single-nucleotide polymorphisms (SNPs) with good imputation quality in HapMap3. To correct for multiple testing bias, the false discovery rate (FDR) correction method was applied, with an FDR q value < 0.05 set as the statistical significance threshold. Among the three analyses, trait pairs showing significant genetic correlations between DM/glycemic traits and asthma were identified. Trait pairs with significance in at least two methods were selected for subsequent analyses.

### 2.4. Genome-Wide Genetic Overlap

Given that genetic correlations reflect only the overall associations across the entire genome, we selected trait pairs showing significance in at least two global genetic correlation analyses and further applied genetic analysis incorporating pleiotropy and annotation (GPA) to explore genome-wide genetic overlap between traits [19]. For each trait pair, GPA classifies SNPs into four models: (i) SNPs not associated with either trait (M00 model), (ii) SNPs associated exclusively with the first trait (M10 model), (iii) SNPs associated exclusively with the second trait (M01 model), and (iv) SNPs associated with both traits (M11 model). This method estimates the proportion of SNPs in each model and evaluates the statistical significance of genome-wide genetic overlap via likelihood ratio tests. The significance threshold was set at a false discovery rate (FDR) q value < 0.05.

### 2.5. Local Genetic Correlations

Global genetic correlations estimate the average shared associations across the entire genome, and these significant correlations may arise from mixed shared associations [20]. Therefore, we further estimated regional genetic correlations via local analysis of covariate association (LAVA). For the LAVA analysis, genotype data from European samples in the 1000 Genomes Project Phase 3 were utilized, and the genome was partitioned into 2495 regions with an average size of 1 Mb. Only regions where SNPs exhibited significance for both traits in the selected trait pairs (*p* < 0.05/2495) were employed to estimate local genetic correlations between the traits.

### 2.6. Identification of Pleiotropic Loci

To identify pleiotropic variants that may influence both traits simultaneously, multiple methods have been employed. First, CPASSOC analysis was performed to calculate pairwise SHet statistics, which are more robust in the presence of heterogeneity [21]. Index SNPs were defined as significant if they met the following criterion: a CPASSOC *p* value < 5 × 10^−8^. Second, to mitigate false-positive results arising from reliance on a single method, we additionally conducted multitrait analysis of GWAS (MTAG). This analysis applies a discrete local maxima approach with multiple testing correction for disease combinations on the basis of GWAS data [22]. SNPs with a PMTAG < 5 × 10^−8^ were identified as genome-wide significant pleiotropic loci. Functional mapping and annotation of genetic associations (FUMA) were used to annotate significant loci identified through cross-trait joint analyses. FUMA provides combined annotation-dependent depletion (CADD) scores and RegulomeDB (RDB) scores. SNPs with CADD scores > 12.37 were considered potentially deleterious variants [23].

### 2.7. Colocalization Analysis

Colocalization is a method used to assess whether two traits share causal genetic variants within a genomic region and is implemented via the COLOC R package (version 5.2.3). A Bayesian approach to compute posterior probabilities, evaluating whether two associated signals (asthma and glucose metabolism traits) are driven by shared causal genetic variants. We performed colocalization analysis on 203 shared SNPs annotated by FUMA between DM or glucose metabolism traits and asthma, extracting variants within a 500 kb window around these SNPs. Five hypotheses were tested in the colocalization analysis, with posterior probabilities calculated for each: Hypothesis 0: No association with either trait; Hypothesis 1/Hypothesis 2: Association with trait 1 or trait 2 only; Hypothesis 3: Association with both traits but driven by two independent causal variants; Hypothesis 4: Associations with both traits driven by a single shared causal variant [24]; and Loci with a posterior probability for Hypothesis 4 (PP.H4) > 0.7 were considered colocalized [25].

### 2.8. Biomarker Expression Level Imputation via Summary-Level Statistics

Proteome-wide association studies (PWAS) have emerged as promising approaches for identifying proteome‒phenotype associations by leveraging genetically inferred proteomic models and GWAS summary statistics. Biomarker expression level imputation using summary-level statistics (BLISS) is a novel method for creating protein imputation models on the basis of summary-level pQTL data. This approach integrates proteomic data with GWAS findings to provide more direct insights into how genetic variants influence disease through alterations in protein abundance and function [26]. Therefore, we systematically investigated complex proteomic associations between glucose metabolism traits and asthma via the BLISS analytical framework. Summary-level pQTL data were sourced from deCODE (n = 35,559) [27] and ARIC (n = 7213) [28]. Proteins with false discovery rate (FDR)-corrected *p* values < 0.05 were considered significant, indicating their potential critical regulatory roles in the pathophysiological interplay between glucose metabolism traits and asthma.

### 2.9. GSMR

Generalized summary-data-based Mendelian randomization (GSMR) constructs a variance‒covariance structure based on correlations among instrumental variables (IVs), which are subsequently used as weights to estimate the overall causal effect via a generalized linear model. This approach enables a more precise evaluation of the impact of genetic factors on complex traits [29]. GSMR employs the heterogeneity in dependent instruments (HEIDI) test to identify and remove IVs exhibiting pleiotropic effects. By eliminating pleiotropic IVs and accounting for linkage disequilibrium (LD), GSMR minimizes false-positive findings and delivers more robust causal inference results.

## 3. Results

### 3.1. Global Genetic Correlation Analysis

**Figure 2:**
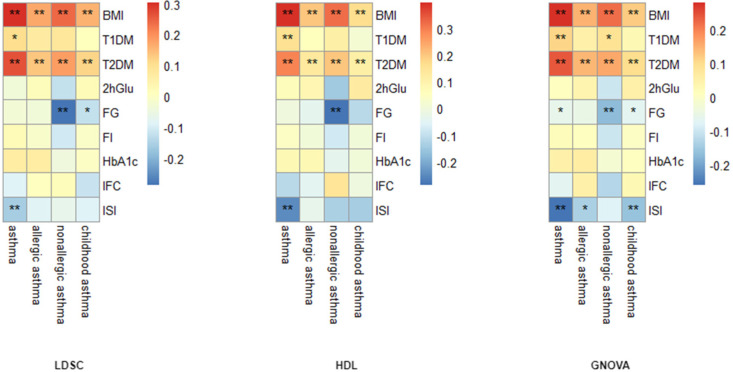
Genetic correlation analysis between asthma phenotypes and diabetes/glycemic traits. *: FDR < 0.05, **: FDR < 0.01.

Global genetic correlation analyses using three methods (LDSC, HDL, GNOVA) revealed significant positive genetic correlations between asthma and both BMI (rg = 0.280–0.397) and T2DM (rg = 0.240–0.289), FDR < 0.05. Specifically, for asthma and BMI, the genetic correlation estimates were 0.304 (LDSC), 0.397 (HDL), and 0.280 (GNOVA) (FDR < 0.05). Asthma and T2DM: The genetic correlation estimates were 0.266 (LDSC), 0.289 (HDL), and 0.24 (GNOVA) (FDR < 0.05). No significant correlations were observed between asthma and other glycemic traits (e.g., fasting glucose/FG, fasting insulin, HbA1c). The details are presented in Figure 2 and Supplementary Table S2.

### 3.2. Genome-Wide Genetic Overlap Analysis

Genome-wide genetic overlap analysis conducted via Pleiotropy and functional Annotation (GPA) included 12 trait pairs with genetic correlations, all of which exhibited significant genetic overlap. The pleiotropy association ratio (PAR), defined as PM 11/(PM 10 + PM 01 + PM 11), was introduced to quantify the proportion of SNPs associated with both traits relative to SNPs associated with at least one trait [30]. GPA revealed the greatest pleiotropy between BMI and asthma (PAR = 0.377), followed by T2DM and asthma (PAR = 0.353). Notably, non-allergic asthma showed stronger genetic overlap with BMI (PAR = 0.332) and T2DM (PAR = 0.304) than allergic asthma (BMI: PAR = 0.219; T2DM: PAR = 0.228) (as detailed in Table 1).

### 3.3. Local Genetic Correlation Analysis

**Figure 3:**
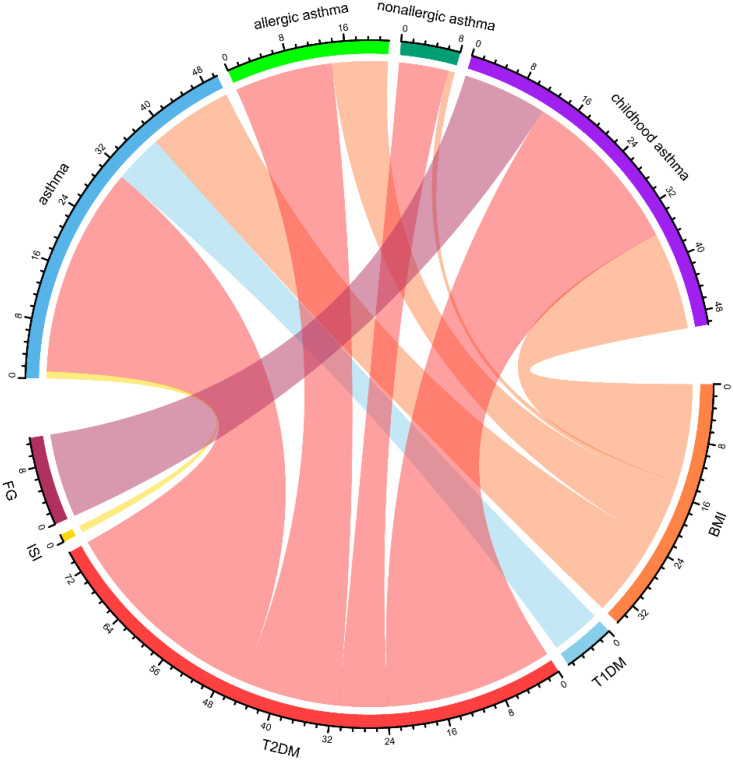
Chord diagram representing the network of local genetic correlations between diabetes and glucose metabolism traits with asthma. A greater width of a ribbon reflects a greater number of loci that are genetically correlated between two phenotypes, highlighting substantial polygenic overlap and suggesting potential pathophysiological mechanisms shared between them. BMI: Body Mass Index, FG: Fasting Glucose, ISI: Insulin Sensitivity Index, T2DM: Type 2 Diabetes Mellitus, T1DM: Type 1 Diabetes Mellitus.

Local genetic correlation evaluates the correlation of genetic effects between two phenotypes within specific genomic regions. This approach provides a refined examination of genetic overlap, enabling the identification of key regions driving shared genetic architecture. After false discovery rate (FDR) correction for the number of tests performed, 111 local regions with both negative and positive local genetic correlations (|rg| = 0.31–0.76, FDR < 0.05) were identified between asthma and diabetes/glucose metabolism traits (BMI, T1DM, T2DM, FG) across distinct phenotypic subgroups (see Supplementary Table S3). Among these, T2DM and asthma presented the greatest number of significant local correlations (70 regions), with the most prominent local genetic correlation observed in region 648 (chr4: 47,408,266–49,325,138). This was followed by BMI and asthma, which showed 32 significant local correlation regions, with the strongest association detected in region 2398 (chr20: 30,569,660–32,484,506) (Figure 3).

### 3.4. GSMR

In the GSMR analysis, DM or glucose metabolism-related traits were first set as exposure factors, and asthma was used as the outcome to identify factors potentially increasing asthma risk. The results demonstrated that elevated BMI significantly increased asthma risk (OR = 1.47, 95% CI: 1.42–1.53). Concurrently, T2DM was also associated with increased asthma risk (OR = 1.06, 95% CI: 1.04–1.08). Reverse-direction GSMR analyses were then performed with asthma as the exposure and DM/glucose metabolism traits as outcomes. These analyses revealed no causal effect of asthma on T2DM or elevated BMI risk. No significant association was observed between T1DM and asthma risk (see Table 2).

### 3.5. Cross-Trait Loci and Causal Variant Analysis

CPASSOC identifies associations across multiple phenotypes and is particularly suited for exploring shared genetic foundations between distinct diseases or traits. MTAG detects shared genetic variants across phenotype pairs by integrating GWAS summary data from paired traits (Trait 1 and Trait 2) to pinpoint loci common to both traits. Through cross-trait joint analyses conducted via MTAG and CPASSOC, followed by annotation via FUMA, we identified SNPs shared across trait pairs. The results revealed the highest number of SNPs shared between asthma and BMI: 139 SNPs for asthma-BMI, 142 for allergic asthma-BMI, 141 for childhood asthma-BMI, and 127 for nonallergic asthma-BMI. For the T2DM-asthma trait pairs, there were 95 (T2DM-asthma), 94 (T2DM-allergic asthma), 85 (T2DM-childhood-onset asthma), and 94 (T2DM-nonallergic asthma) shared SNPs. To infer shared causal SNPs, colocalization analysis was subsequently performed on these SNPs across traits via the COLOC algorithm. This process identified 24 causal variants shared between diabetes/glucose metabolism traits and asthma, which were predominantly located on chromosome 8 (Supplementary Table S4).

### 3.6. Identification of Proteins Associated with Comorbid Glucose Metabolism Traits and Asthma

**Figure 4:**
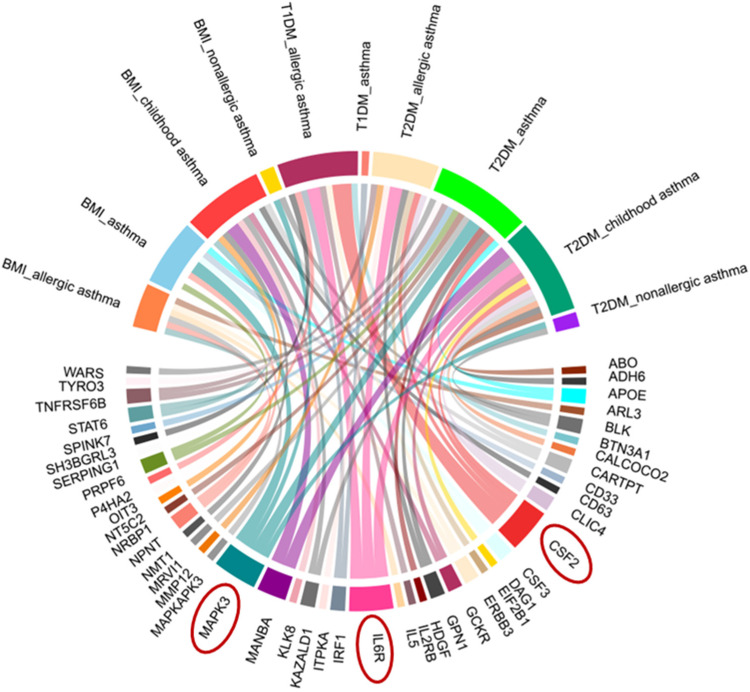
Chord diagram of proteins associated with glucose metabolism traits and asthma comorbidity identified by BLISS. Red circles highlight key proteins (MAPK3, IL6R, CSF2) with prominent associations.

**Figure 5:**
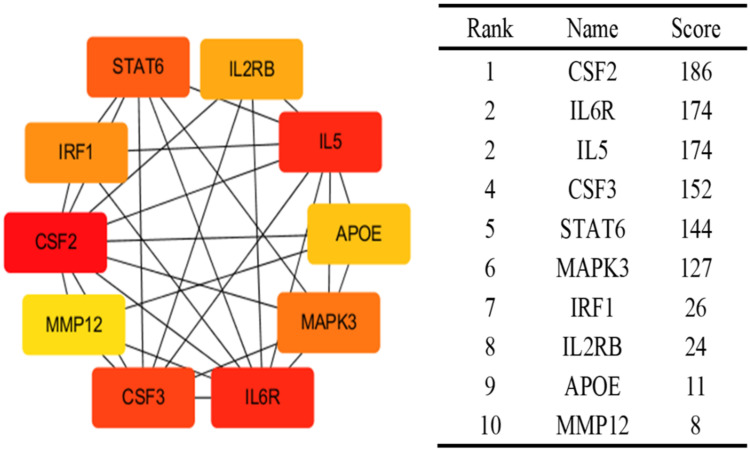
Top 10 proteins associated with both glucose metabolism traits and asthma identified by Cytohubba. The colors intensity represents the strength of the MCC correlation between the proteins.

**Figure 6:**
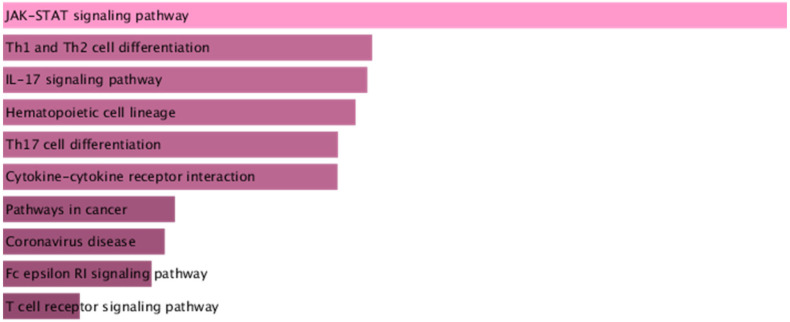
KEGG enrichment analysis of the core proteins associated with glucose metabolism traits and asthma. The color gradient of the bars represents the statistical significance (*p*-value) of the KEGG pathway enrichment.

**Figure 7:**
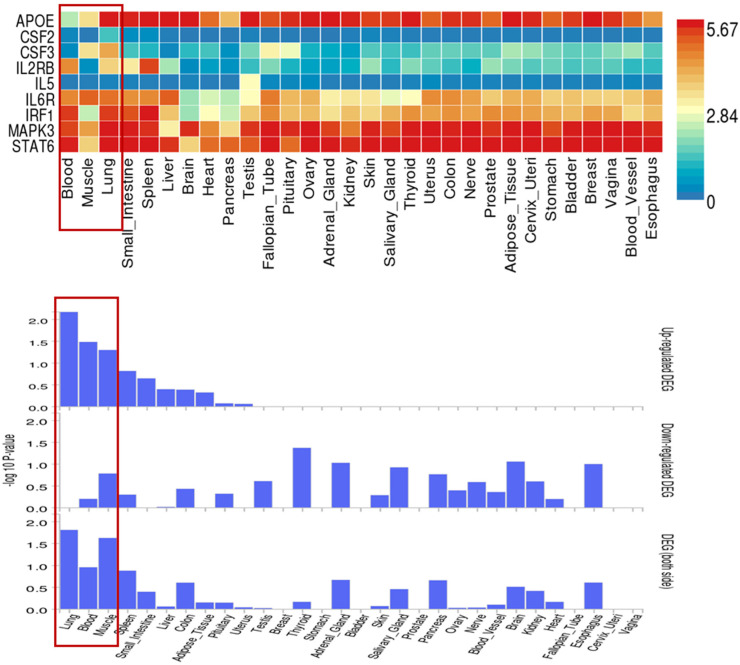
Tissue-specific expression of 10 core proteins related to glucose metabolism traits and asthma in 30 general tissue types. Red boxes highlight three key tissues (Blood, Skeletal Muscle, Lung) and their corresponding profiles of core protein expression levels and statistical significance, which are closely associated with the comorbidity of glucose metabolism traits and asthma.

Using the BLISS method, we identified 46 proteins associated with comorbid risks of glucose metabolism traits and asthma. Among these proteins, MAPK3, IL6R, and CSF2—the most widely shared proteins—emerged as potential key regulators influencing the comorbidity of glucose metabolism dysregulation and asthma (Supplementary Table S5, Figure 4). Protein‒protein interaction (PPI) networks for the identified shared proteins were constructed via the STRING database. The CytoHubba plugin on the Cytoscape (version 3.10.1) platform, which employs the maximal clique centrality (MCC) algorithm, was used to screen the top 10 hub proteins on the basis of network topology weights. These core hub proteins included CSF2, IL6R, IL5, CSF3, STAT6, MAPK3, IRF1, IL2RB, APOE, and MMP12 (Figure 5). The 10 hub proteins were subsequently uploaded to the Enrichr web tool (https://maayanlab.cloud/Enrichr/) [31] for KEGG pathway enrichment analysis, revealing their predominant involvement in the JAK-STAT signaling pathway, Th1 and Th2 cell differentiation, and the IL-17 signaling pathway (Figure 6). Further tissue enrichment analysis via FUMA demonstrated that these proteins were predominantly enriched in lung, blood, and muscle tissues (Figure 7).

## 4. Discussion

Diabetes and asthma represent two prevalent chronic diseases that impose significant health burdens globally. Accumulating clinical evidence indicates a positive association between diabetes and asthma, with chronic inflammation being currently recognized as a shared pathophysiological mechanism [32]. However, the underlying shared genetic architecture remains poorly understood. Through leveraging GWAS summary statistics and employing a series of novel bioinformatics approaches, we elucidated the shared genetic architecture linking diabetes, glycometabolic traits, and asthma. Our findings demonstrate a causal role for obesity and T2DM in elevating asthma risk, highlighting the impact of metabolic dysfunction on airway inflammation, thereby providing critical insights into the genetic foundations of these comorbid diseases. Key proteins central to JAK-STAT signaling, notably IL6R and CSF2, may mediate this interplay. Collectively, these results provide crucial insights into the genetic underpinnings of this comorbidity.

To our knowledge, this study represents the first systematic investigation of the shared genetic architecture between diabetes mellitus (T1DM/T2DM) and glycometabolic traits and asthma subtypes (allergic asthma, nonallergic asthma, and childhood-onset asthma). Utilizing multiple global genetic correlation methods, we identified significant genetic correlations between BMI, T2DM, and asthma phenotypes. These results suggest that obesity and T2DM may influence asthma pathogenesis through shared genetic mechanisms, such as inflammatory regulation or insulin resistance pathways [33]. GPA analysis further confirmed significant genetic overlap across all trait pairs, with differences in pleiotropy association ratio (PAR) values indicating varying degrees of genetic sharing among asthma subtypes. For instance, the highest PAR between BMI and asthma implies greater shared pleiotropic loci, potentially driven by proinflammatory cytokines released from adipose tissue that directly exacerbate airway inflammation in asthma [34].

Local genetic correlation analysis identified the most significant regions between T2DM and asthma (70 regions), followed by BMI and asthma (32 regions). Genome partitioning revealed significant local genetic correlations in specific genomic regions (e.g., chr1: 38,474,037–40,200,950), indicating not only shared genetic etiology (i.e., biological pleiotropy) but also suggesting potential causal relationships (i.e., vertical pleiotropy) [35]. Moreover, cross-trait analyses pinpointed 24 candidate pleiotropic variants across the 12 genetically correlated trait pairs involving diabetes/glycemic traits and asthma.

Subsequent causal inference analyses established unidirectional causal effects of BMI (OR = 1.47) and T2DM (OR = 1.06) on asthma risk, whereas reverse-direction GSMR revealed no evidence of asthma increasing metabolic dysregulation or diabetes risk. For T2DM, hyperglycemia-induced advanced glycation end products (AGEs) may increase airway hyperresponsiveness via receptor-mediated oxidative stress (RAGE signaling) [36]. Conversely, the lack of reverse causality (asthma towards T2DM/BMI) suggests that asthma treatments, such as corticosteroids, do not directly worsen metabolic dysfunction, although confounding by factors like physical inactivity requires further exploration. Notably, we found no significant causal association between T1DM and asthma. This indicates that despite partial shared genetic backgrounds (e.g., HLA regions), the primary pathogenic pathways for T1DM and asthma likely diverge.

We systematically dissected shared genetic effects by integrating global genetic correlation methods (LDSC, HDL, and GNOVA) with pleiotropic locus identification tools (MTAG, CPASSOC, and COLOC). PWAS identified IL6R, MAPK3, and CSF2 as the proteins most extensively shared between glycemic traits and asthma. IL6R gene encodes a subunit of the interleukin-6 receptor. Consistent with our findings, a Mendelian randomization study investigating the relationship between IL6R signaling pathway blockade and respiratory disease risk demonstrated that IL6R inhibition is associated with reduced asthma risk [37]. Meta-analyses confirm significantly elevated serum IL-6 levels in asthma patients, correlating with disease severity, exacerbation frequency, and impaired lung function [38], while IL6R inhibition also demonstrates therapeutic benefits in T2DM patients [39]. CSF2, also known as granulocyte‒macrophage colony‒stimulating factor (GM-CSF), is a pleiotropic cytokine produced by diverse cell types, including T cells, B cells, macrophages, and fibroblasts. This cytokine binds to its receptor, activating JAK2 kinase and triggering downstream signaling cascades, such as the JAK-STAT pathway, alongside the MAPK and PI3K pathways [40]. CSF2/GM-CSF exerts core regulatory functions primarily through JAK-STAT signaling. The activation of transcription factors (e.g., STAT3) regulates immune cell proliferation and differentiation [40]. Modulation of inflammation-related gene expression coordinates innate and adaptive immune responses [41]. GM-CSF enhances dendritic cell antigen capture and presentation, thereby activating Th2/Th17 immune cascades. These effects mediate the chemotactic recruitment of pulmonary neutrophils and promote allergic immunopathology in chronic lung diseases [42]. KEGG enrichment analysis revealed significant enrichment of core proteins associated with glucose metabolism traits and asthma in the JAK-STAT signaling pathway, Th1/Th2 differentiation pathway, and IL-17 signaling pathway. These findings implicate these pathways as critical mediators in the co-occurrence of diabetes and asthma.

Despite the robust genetic methods employed in our study, several limitations should be acknowledged. First, the reliance on European ancestry data limits the generalizability of our findings to other populations, where genetic and environmental interactions may differ. Future studies should validate these findings in multiethnic cohorts. Second, functional validation of candidate loci is needed to confirm mechanistic links. Third, the role of epigenetics in mediating gene‒environment interactions remains unexplored. Further research is warranted to address these limitations and to provide a more comprehensive understanding of the shared genetic architecture between diabetes and asthma.

## Figures and Tables

**Table 1:** Genetic Overlap Estimates for 12 Trait Pairs Diabetes/Glycemic Traits.

Trait Pairs	PM 11	PAR	*p* Value
BMI–allergic asthma	0.100	0.219	<0.001
T2DM–allergic asthma	0.086	0.228	<0.001
BMI–childhood asthma	0.079	0.176	<0.001
FG–childhood asthma	0.012	0.077	<0.001
T2DM–childhood asthma	0.072	0.196	<0.001
BMI–non-allergic asthma	0.165	0.332	<0.001
FG–non-allergic asthma	0.014	0.048	4.21 × 10^−34^
T2DM–non-allergic asthma	0.132	0.304	<0.001
BMI–asthma	0.189	0.377	<0.001
T1DM–asthma	0.043	0.143	<0.001
ISI–asthma	0.033	0.111	9.66 × 10^−202^
T2DM–asthma	0.155	0.353	<0.001

**Table 2:** Bidirectional GSMR analysis of diabetes/glycemic traits and asthma.

Exposure	Outcome	OR	Lower 95%CI	Upper 95%CI	FDR	nsnp
BMI	Asthma	1.47	1.42	1.53	5.46 × 10^−92^	1369
T2DM	Asthma	1.06	1.04	1.08	2.54 × 10^−11^	1101
FG	Asthma	0.88	0.81	0.97	4.30 × 10^−2^	115
FI	Asthma	0.68	0.56	0.83	7.13 × 10^−4^	37
HbA1c	Asthma	0.98	0.85	1.12	8.80 × 10^−1^	111
2hGlu	Asthma	1.03	0.96	1.11	8.80 × 10^−1^	15
T1DM	Asthma	1.01	1.00	1.03	2.20 × 10^−1^	121
IFC	Asthma	1.24	1.04	1.49	6.94 × 10^−2^	5
Asthma	BMI	1.01	1.00	1.03	3.16 × 10^−1^	29
Asthma	T2DM	1.02	0.99	1.05	8.07 × 10^−1^	31
Asthma	FG	0.99	0.98	1.00	8.07 × 10^−1^	36
Asthma	FI	1.00	0.98	1.01	1.00	36
Asthma	HbA1c	1.00	0.99	1.01	1.00	36
Asthma	2hGlu	0.99	0.94	1.05	1.00	36
Asthma	T1DM	1.06	0.96	1.18	1.00	34
Asthma	IFC	0.97	0.93	1.01	8.07 × 10^−1^	37
Asthma	ISI	1.02	0.98	1.07	1.00	37

## Data Availability

All the data generated or analyzed during this study are included in its additional files.

## Supplementary Materials

The following supporting information can be downloaded at: https://www.mdpi.com/article/10.3390/children12111443/s1, Table S1: Sources of GWAS Data; Table S2: Genetic Correlations Between Diabetes/Glycemic Traits and Asthma Phenotypes; Table S3: Local Colocalization Analysis of Diabetes and Glucose Metabolism Traits with Asthma Subtypes; Table S4: Colocalization Loci Identified by Colocalization Analysis of Pleiotropic Loci; Table S5: Proteins associated with glucose metabolism traits and asthma comorbidity identified by BLISS.

## Abbreviations

The following abbreviations are used in this manuscript:

AGEs	Advanced Glycation End Products
AHR	Airway Hyperresponsiveness
BMI	Body Mass Index
BLISS	Biomarker Expression Level Imputation using Summary Statistics
COLOC	Colocalization
CPASSOC	Cross-Phenotype Association
DM	Diabetes Mellitus
FDR	False Discovery Rate
FG	Fasting Glucose
FI	Fasting Insulin
FUMA	Functional Mapping and Annotation of Genetic Associations
GNOVA	Genetic Covariance Analysis
GSMR	Generalized Summary-Data-Based Mendelian Randomization
GWAS	Genome-Wide Association Study
HDL	High-Definition Likelihood Analysis
HEIDI	Heterogeneity in Dependent Instruments
ICS	Inhaled Corticosteroids
IFC	Insulin Fold Change after Oral Glucose Tolerance Test
ISI	Insulin Sensitivity Index
LABA	Long-Acting Beta-Adrenoceptor Agonists
LD	Linkage Disequilibrium
LDSC	Linkage Disequilibrium Score Regression
LAVA	Local Genetic Variant Association Analysis
MCC	Maximal Clique Centrality
MTAG	Multitrait Analysis of GWAS
PAR	Pleiotropy Association Ratio
PPI	Protein‒Protein Interaction
PWAS	Proteome-Wide Association Study
RAGE	Receptor for Advanced Glycation Endproducts
SNPs	Single Nucleotide Polymorphisms
T1DM	Type 1 Diabetes Mellitus
T2DM	Type 2 Diabetes Mellitus
2hGlu	2 Hour Postprandial Glucose
